# Composition of weekly physical activity in adolescents by level of physical activity

**DOI:** 10.1186/s12889-020-08711-8

**Published:** 2020-04-25

**Authors:** Dorota Groffik, Karel Fromel, Petr Badura

**Affiliations:** 1 Institute of Sport Sciences, The Jerzy Kukuczka Academy of Physical Education, Katowice, Poland; 2 Faculty of Physical Culture, Palacký University Olomouc, Olomouc, Czech Republic

**Keywords:** Physical activity, Step count, Day of the week, Adolescents, Pedometer

## Abstract

**Background:**

Physical activity (PA) in adolescence is crucial for lifelong healthy lifestyle, and attention is needed to adolescents at health risk due to insufficient PA. This study investigates the composition of weekly PA in adolescents by PA level and provides a rationale for change in their lifestyles.

**Methods:**

The research was conducted at 66 schools in Poland between 2009 and 2016, among 949 girls and 650 boys aged 15–18 years. We used pedometers to monitor weekly PA with data assessed using the Indares web app. The participants were split into three groups by mean daily step count (lower, < 9000; middle, 9000–12,999; and higher, ≥ 13,000 steps/day), as a reliable and non-expensive indicator of PA.

**Results:**

We did not observe statistically significant differences in composition of 7-day PA between participants with varying levels of PA, regardless of their gender (*F*_(12,9558)_ = 0.60; *p* = 0.841; *η*_*p*_^*2*^ > 0.000). The smallest differences in daily step counts by PA level were found on Mondays and the biggest on Fridays and Saturdays, in boys and girls; the differences between average school and average weekend days were most pronounced in less active girls (1677 steps/day) and boys (1886 steps/day). During the school week, the highest proportion of less active girls met the 11,000 steps/day recommendation on Fridays (21.9%), statistically significantly less than on other school days (*p* < 0.001). Similarly, less active boys (22.2%) had significantly less activity on Fridays than on other school days, except for Mondays (*p* = 0.143) Analogous pattern was apparent also in more active adolescents.

**Conclusions:**

Less active adolescents have comparable composition of weekly PA to the more active ones but they can hardly meet the generally accepted PA recommendations. Better understanding of weekly PA composition and rates of meeting PA recommendations by day of the week can lead to more efficient interventions improving lifestyles. The recommendation of 9000 steps/day most days of the week, thus, appears appropriate for less active adolescents, as a motivating achievable goal.

## Background

Among children and adolescents, there is a serious risk of obesity [1], cardiovascular disease [2], metabolic [3], and mental disorders [4]. Rapid decreases in physical activity (PA) occur during adolescence [5–8] and continue later, in adulthood [9, 10]. Similarly, there is a decline in level of vigorous PA [11, 12] and growing lack of interest in physical education lessons in adolescence [13]. Boys have been shown to be more physically active than girls during both school and weekend days [14–16], while adolescents of both genders are less physically active on weekends than on school days [17–19]. This is confirmed by results from Central Europe, with the most critical day for PA being Sunday, as shown in adolescents from Poland, the Czech Republic and Slovakia [20].

The majority of adolescents in Central Europe do not meet the moderate-to-vigorous physical activity recommendation of 60 min a day or step count recommendations [8, 21–25]. Indeed, in 2012, only 35 of 131 European studies reported that youth were meeting the daily PA recommendation [26]. Given that PA in adulthood is linked to PA in adolescence, this period of life is critical for PA promotion [27, 28]. Increase in PA among Central and East European adolescents is also essential because an alarming amount of time spent being sedentary was reported from these countries [22, 29, 30].

Insufficient physical activity, together with excessive sitting time, is a leading risk factor for non-communicable diseases and has a negative effect on mental health and quality of life [31]. Adolescents who maintain a high level of PA in their young adulthood are at significantly lower risk of cardiovascular disease and are more likely to maintain good mental health status compared to adolescents with consistently low PA [32]. For these reasons, the at-risk adolescents typified by lack of PA, marked physical inactivity, and excessive sitting time deserve special attention. Therefore, we have long investigated the following research questions: what is the weekly composition of PA in adolescents with generally low levels of PA, and how should school environment reflect potential specific features of students’ weekly PA composition in the school lifestyle?

The aim of the present study was then to determine differences in the composition of weekly PA between less and more physically active adolescent boys and girls and to identify potential stimuli for change in lifestyle among adolescents at highest health risk.

## Methods

### Participants and setting

The research was conducted annually between 2009 and 2016 at 66 secondary schools (7–10 schools each year) in the Katowice region of Poland. To select the schools, we used the quota sampling based on the following criteria: place of residence of the student teacher, size of city, type of school (grammar, vocational, and professional secondary schools), first participation in research of this sort, and consent of school administration. All the schools in the sample came from the Silesia Province with almost 5 million inhabitants and the socioeconomic level slightly below European average. Specialized schools (such as sports-focused schools) were not included in the sample.

In total, 1599 adolescents (949 girls and 650 boys) agreed to participate (Table 1); those who had regular swimming training sessions (not possible to be monitored by pedometers) or were engaged in sports disallowing them from taking part in additional training or competition with pedometer were not included in subsequent analyses (every year, 8–13 participants). Our sample consisted of 141 boys and 207 girls aged fifteen, 196 boys and 167 girls aged sixteen, 190 boys and 289 girls aged seventeen, and 123 boys and 286 girls aged eighteen years. All the participants reported to be of Polish nationality. On average, 5–8% of students refused to participate at each school. In all, 11% of boys and 6% of girls were classified as obese.

**Table 1:** Sample characteristic

Gender/level of PA	N	Age (Years)		Weight (kg)		Height (cm)		BMI (kg·m^− 2^)		Steps/day	
		M	SD	M	SD	M	SD	M	SD	M	SD
Boys-lower	230	16.68	0.97	68.90	12.99	177.10	6.83	21.92	3.71	7168	1399
Boys-middle	248	16.64	0.89	66.75	10.83	177.13	7.50	21.23	2.88	10,755	1106
Boys-higher	172	16.31	0.84	66.91	11.74	176.76	8.30	21.32	2.85	15,716	2299
Girls-lower	375	17.00	0.91	56.93	8.93	166.47	6.23	20.52	2.88	7238	1287
Girls-middle	422	16.77	0.95	56.54	7.96	165.79	5.86	20.54	2.45	10,845	1117
Girls-higher	152	16.70	0.95	57.99	9.20	166.73	6.31	20.80	2.61	15,281	2163

*M* Mean, *SD* Standard deviation, *BMI* Body mass index, lower (mean < 9000 steps/day), middle (mean = 9000–12,999 steps/day), higher (mean ≥ 13,000 steps/day)

Girls and boys were split into categories by their level physical activity (i.e. mean step count per day), classified in line with Adams et al. [33] and Tudor-Locke et al. [34]: lower (mean < 9000 steps/day); middle (mean = 9000–12,999 steps/day); and higher (mean ≥ 13,000 steps/day). The respondents were further split by age as younger (aged 15–16 years) vs. older (aged 17–18 years), with the composition of the sample by the level of their PA was as follows: younger boys (107 lower, 117 middle, and 113 higher PA), older boys (123 lower, 131 middle, and 59 higher PA), younger girls (119 lower, 185 middle, and 70 higher PA) and older girls (256 lower, 237 middle, and 82 higher PA).

### Measures

Initial meeting with participants took place in ICT classrooms, so as not to intervene with common school program. All the participants registered using the web application called Indares (www.indares.com). The Tanita UM-075 (Tanita, Tokyo, Japan) was used to measure participants’ weight and the Leicester Height Measure Mk II (Invicta Plastics, Leicester, United Kingdom) to measure their height. We instructed students in PA monitoring, entering the data in the Indares application. The Digi-Walker SW-700 (Yamax Co., Yasama Corp., Tokyo, Japan) was used to measure PA. The pedometers were calibrated by the research team under habitual locomotive conditions (slow- and fast-walking) with a tolerable deviation up to 5%. The students were trained on how to wear the pedometers (at the hip bone on the right side of the waist) and when to take them off (swimming, showering, sleeping). Each student his/her achieved number of steps each evening. The participants were instructed to reset their pedometers at the start of each day, and wore pedometers for seven consecutive days starting from the day of introductory meeting (if the initial meeting was held on Friday, the first day of actual monitoring was postponed until Monday). To minimise bias by, for instance, weather conditions on a particular day, the start of the monitoring at school was relatively equally distributed across the 5 days of a week; the monitoring was never launched on a weekend day.

### Data processing

The pedometer data were evaluated in line with the recommendations of Craig et al. [35]. Step counts outside the 1000–30,000 steps/day range were truncated to these respective values. Records containing at least three school days and one weekend day were considered valid. Missing data were replaced by data from the previous day. If the data for first school or weekend day were missing, then the values of the following day were used. Overall, we excluded 135 participants from the analyses and replaced data on daily step count for 189 days.

### Statistical analyses

The data were analysed using Statistica version 13 (StatSoft, Prague, Czech Republic). We used descriptive statistics to provide basic information on the study sample. Arithmetic means, standard deviations and percentages derived from frequency analyses and cross tables were used to assess the differences between groups of participants in rates of meeting the daily step count recommendations. The interaction effect of weekly PA composition by days of the week, by gender. by levels of PA and the study period was analysed using repeated-measures ANOVA (7 days × 3 level of PA × 2 gender categories); Box’s M test and Mauchly’s sphericity test were used to determine whether the ANOVA assumptions were violated. To assess the differences between PA on specific days of week in boys and girls separately, we used one-way ANOVA. The differences between school days and weekend days by level of PA were assessed using the dependent-samples t-test, and variation in step counts on school days by sign test. The effect size was estimated using coefficients of (0.01–0.059 small effect size, 0.06–0.139 medium effect size, and ≥ 0.14 large effect size) and *d* (0.2–0.49 small effect size, 0.5–0.79 medium effect size, and ≥ 0.8 large effect size) [36, 37]. As a form of sensitivity analysis, we compared the composition of weekly PA in adolescents (split by gender and level of their PA) separately between 2009 and 2012 and 2013–2016. This analysis yielded almost identical pattern of PA composition over a week – 2009-2013 (*F*_(12,4830)_ = 0.56; *p* = 0.878; *η*_*p*_^*2*^ = 0.001) 2013–2016 (*F*_(12,4629)_ = 1.08; *p* = 0.377; *η*_*p*_^*2*^ = 0.003) – regardless of gender and level of respondents’ PA, so we present the data for the entire 8-year period. The level of statistical significance, α, was a priori set at 5%. Practical significance in weekly PA was set at a difference of 2000 steps/day. Regarding the weekly PA recommendation, it was 10%.

## Results

### Weekly physical activity (steps/day) composition in adolescents by gender

**Fig. 1:**
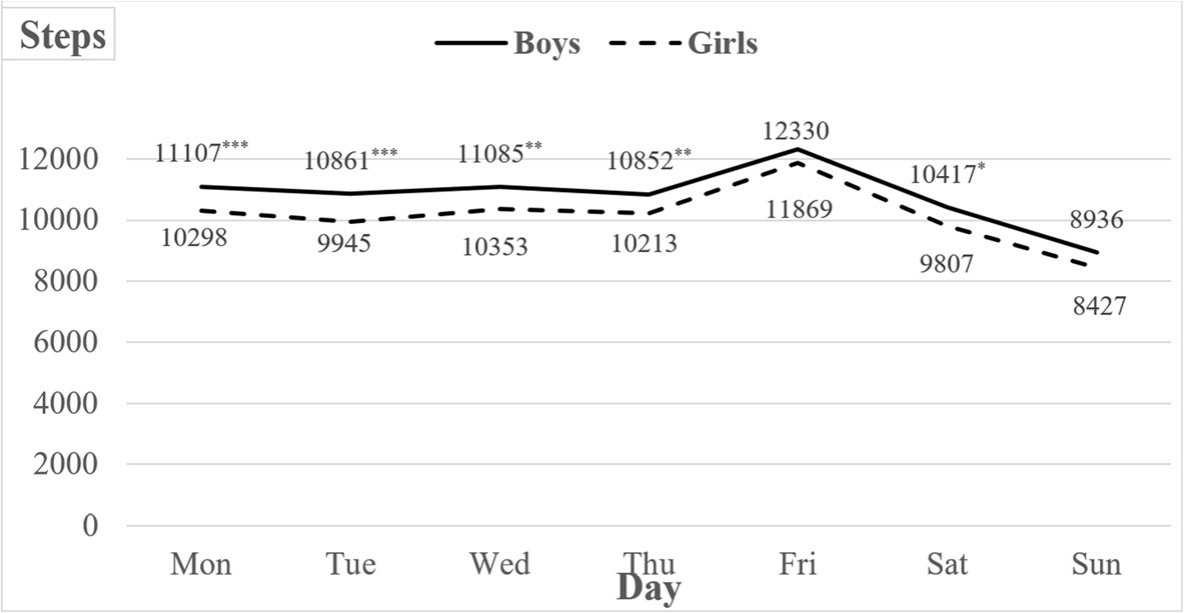
The daily step counts for boys and girls by the day of the week.. ^***^p < 0.001;^**^p < 0.01; ^*^p < 0.05, indicating statistical significance in difference of step counts on specific days of the week between boys and girls

On average, we observed girls and boys to reach 10,130 ± 3121steps/day and 10,799 ± 3692 steps/day, respectively. The differences in daily step counts by day of the week were of statistical significance in both girls and boys (*F*_(6,9582)_ = 98.76; *p* < 0.001; *η*_*p*_^2^ = 0.058) (Fig. 1). Regardless of gender, the participants reached significantly lower step counts on Sundays compared with other days of the week (*p* < 0.001). On the contrary, Friday was the day with significantly higher step counts, again for both girls (*p* < 0.001) and boys (*p* < 0.01).

### Weekly physical activity (steps/day) composition by level of PA (lower, middle and higher) in boys and girls

**Fig. 2:**
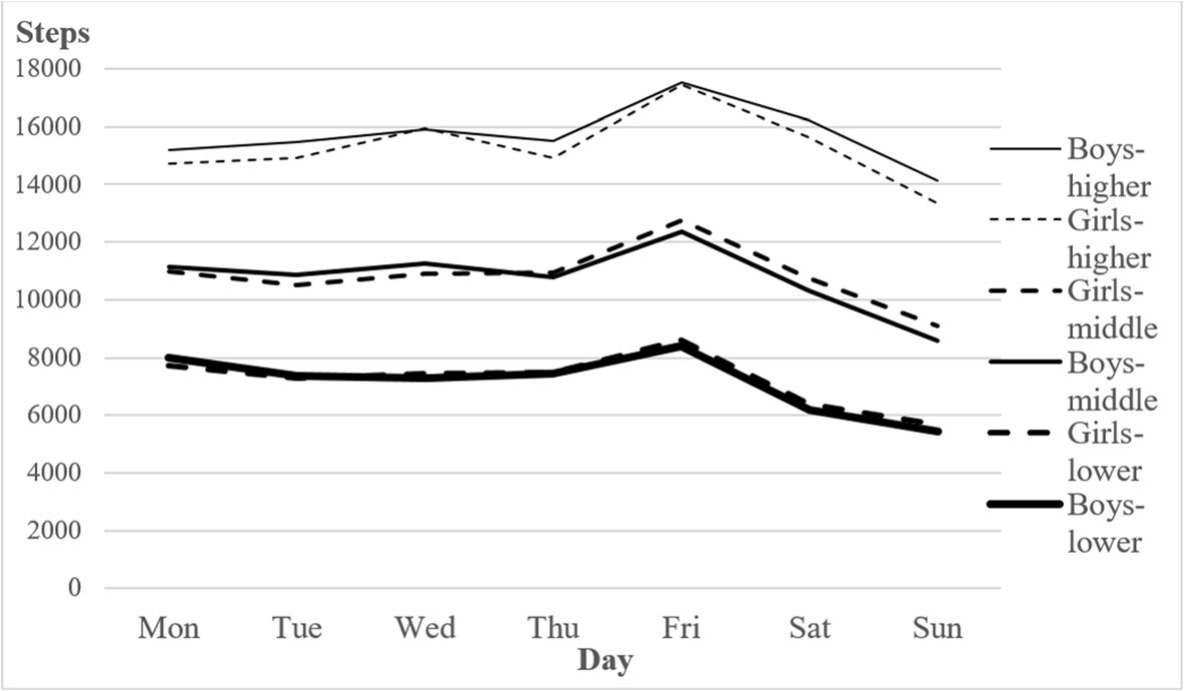
Composition of weekly physical activity (steps/day) by gender and overall level of physical activity (lower – mean < 9000 steps/day, middle – mean 9000–12,999 steps/day, higher – mean ≥ 13,000 steps/day)

Analysis of interaction effects showed that the differences in weekly PA composition were not statistically significant across different levels of PA, in both genders and regardless of day of the week [day × gender × PA level] (*F*_(12,9558)_ = 0.60; *p* = 0.841; *η*_*p*_^*2*^ > 0.000), as is apparent in Fig. 2. The lowest differences in daily step count between girls with lower level of PA and girls with higher level of PA (6995 steps/day) were found on Mondays. Boys with lower level of PA recorded the least pronounced difference from those with higher level of PA on Mondays too (7207 steps/day). On the contrary, the largest differences were observed on Saturdays (9233 steps/day in girls; 10,027 steps/day in boys). Inclusion of the age factor (younger vs. older respondents) did not statistically significantly influence the composition of weekly PA among groups with different PA levels [days × gender × age × PA level] (*F*_(12,9522)_ = 1.38; *p* = 0.166; *η*_*p*_^*2*^ > 0.002). Similarly, the composition of weekly PA did not differ significantly by level of PA when the study period (two-year stages) was taken into account [days × gender × study period × PA level] (*F*_(36,9450)_ = 0.96; *p* = 0.532; *η*_*p*_^*2*^ > 0.004). Interestingly, we observed an increase in PA, expressed by steps/day, over the 8-year study period. [days × gender × study period] (*F*_(18,9546)_ = 2.86; *p* < 0.001; *η*_*p*_^*2*^ > 0.005). However, this increase was not detected in the least physically active boys and girls. [gender × study period] (*F*_(3,597)_ = 0.52; *p* = 0.669; *η*_*p*_^*2*^ > 0.003).

### Comparison of mean daily step counts between school and weekend days in boys and girls with different level of physical activity

The difference between average school and weekend days was most pronounced in girls in with lower level of PA – 1677 steps/day (mean 7717 steps/day in school days and 6040 steps/day in weekend). Similarly, it was the most marked in the least active group of boys – 1886 steps/day (mean 7707 steps/day in school days and 5821 steps/day in weekend) and reached statistical significance (Table 2). This school day–weekend pattern held true for both boys and girls across the levels of their PA, with a single exception. The difference between school and weekend days lacked statistical significance in of boys with higher level of PA.

**Table 2:** The differences in mean steps/day between school and weekend days by level of physical activity in girls and boys

Gender	Level of physical activity	n	School days		Weekend		t	p	d
			M	SD	M	SD			
Boys	lower	230	7707	1669	5821	2624	9.14	< 0.001	0.575^a^
	middle	248	11,280	1663	9444	3346	6.57	< 0.001	0.398^a^
	higher	172	15,935	2600	15,169	5050	1.74	0.843	0.127
Girls	lower	375	7717	1543	6040	2361	11.47	< 0.001	0.565^a^
	middle	422	11,216	1542	9918	2902	7.13	< 0.001	0.331^a^
	higher	152	15,600	2531	14,485	4026	2.92	0.004	0.226^a^

*M* Mean, *SD* Standard deviation, *t* Dependent sample test, *p* Level of significance, *d* – Cohen’s effect size coefficient, ^a^small effect size, lower – mean < 9000 steps/day, middle – mean 9000–12,999 steps/day, higher – mean ≥ 13,000 steps/day

### Meeting the 11,000 steps/day (9000 steps/day) recommendation on days of the week by level of physical activity in boys and girls

**Fig. 3:**
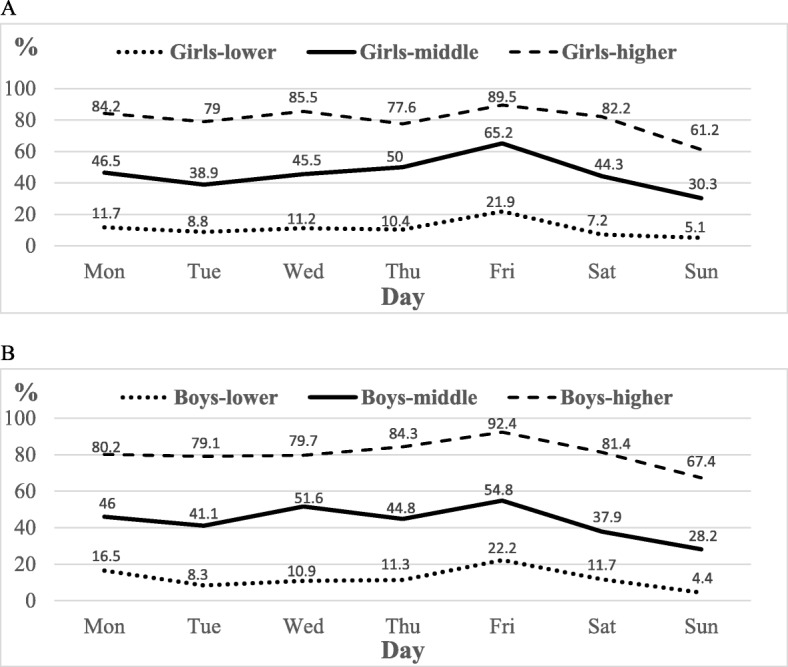
Girls’ (**a**) and boys’ (**b**) rates of meeting the 11,000 steps/day recommendation per day of the week by level of physical activity (lower – mean < 9000 steps/day, middle – mean 9000–12,999 steps/day, higher – mean ≥ 13,000 steps/day)

Logically, adolescents’ rate of meeting the 11,000 steps/day recommendation on specific days of the week by level of PA corresponded to the weekly composition of step count values (Fig. 3a, b). The lowest rates of girls and boys in all three PA groups met the recommendation on Sundays, which was significantly low compared with other days of the week (*Z* = 2.18–9.22; *p* = 0.029–0.000; *η*^*2*^ = 0.028–0.201). On school days, the highest rate of the less active girls met the recommendations on Fridays (21.9%), statistically and practically different from all other days (*p* < 0.001). This was similar in boys, apart from lack of a significant difference between Fridays and Mondays (16.5%) (*p* = 0.143).

Compared with the 11,000 steps/day recommendation, less active adolescents would be slightly more successful in achieving 9000 steps/day, if this less strict boundary was set. The rates of meeting this hypothetical less severe recommendation were as follows: Monday – boys 31.3% and girls 30.4%; Tuesday – boys 25.6% and girls 24.0%; Wednesday – boys 24.8% and girls 25.6%; Thursday – boys 25.7% and girls 29.9%; Friday – boys 41.7% and girls 41.1%; Saturday – boys 18.3% and girls 20.3%; and Sunday – boys 11.7% and girls 14.1%.

## Discussion

The key finding of the present study highlights the fact that the weekly composition of PA is analogous in more and less physically active girls and boys. This implies that girls and boys who accumulate less PA respond to varying in-school and out-of-school conditions on specific days of the week in a similar way to physically active adolescent populations. The lowest difference in daily step count between boys and girls with higher and lower PA was found on Mondays; efforts to decrease this difference even further might be especially important as a way to compensate for Sundays, as the most critical day with the lowest level of PA. The low level of PA on Sundays has been previously reported by numerous studies [20, 38–41]. The unfavourable PA on Sundays in the least physically active adolescents has not been getting any better thus far. One of the main causes lies likely in the less tight daily schedule, compared with school days. School-based PA represents 33 and 34% of total daily PA in Polish girls and boys, respectively [42], thus absence of this setting is to a large extent attributable for the low PA level on weekends. Extending the offer of popular organized physical activities, with competitions usually taking place on weekends, supported by successful interventions, could be beneficial. Increase in Monday PA in less physically active adolescents may also distort the consistently low level of PA among this population on other days of the week. These findings should be respected in the offer of physical activities for less physically active adolescents. This holds true for school programs, in-school extracurricular activities, comprehensive physical activity programs, but also for clubs and other institutions providing extracurricular physical activities. Such an approach would also require to develop a system of identification of at-risk adolescents, complemented by diagnostics of sport or physical activity preferences.

Mean daily step count on school days and weekends in less active girls equals 7717 steps/day (6040 steps/day at weekends), and in boys, 7707 steps/day (5821 steps/day at weekends); these low counts are alarming. Our study confirmed again that girls respond better to wearables than boys [20, 43] (e.g. less missing data, interest in use of pedometers after the end of study period) and that the gender-differences in PA volume (as expressed by step counts) have been diminishing. According to [44], the use of pedometers in less active adolescents might lead to an increase of roughly 2500 steps/day. In the current era of ubiquitous technologies, application of easy-to-use fitness trackers or mobile phone apps to track and motivate for PA could promote PA in adolescents [45, 46].

Given recent trends in adolescents’ PA, one can infer that the most widespread recommendations of 10,000 steps/day [47, 48], 10,000–11,700 steps/day [34], 12,000 steps/day [49, 50, 51], the 11,000 steps/day for both boys and girls promoted by our team [41, 52, 53], or 13,000–16,000 steps/day focused on weight-reduction behaviours [54], are not accepted well by adolescents with lower levels of PA. In Canada, Colley et al. [50] suggested revising the recommendation from 13,500 to 12,000 steps/day among 6–19-years-old children and adolescents, which they found more appropriate benchmark for assessing the guideline of 60 min of daily moderate-to-vigorous PA. It needs to be further verified, if the recommended combination of goal-setting and walking exercise program [55] works also for less physically active adolescents.

The pedometer-friendly value of 9000 steps/day, proposed by Adams et al. [33], appears to be the most feasible for less physically active adolescents, while still being motivational. The recommended value of 9000 steps/day for adolescents is adequate given the number of <5000 steps/day as a step-defined sedentary lifestyle index for adults [56]. Unfortunately, we lack evidence that would enable us to set a step-defined sedentary lifestyle index in children or adolescents. Moreover, it remains unknown if the finding of Anson and Madras [57] – that university students with higher daily goals for steps reached higher step counts than those with lower goals – is applicable also to adolescents. We assume that feelings of satisfaction gained by meeting the 9000 as opposed to the 11,000 steps/day (which we observed in 13.4% of boys and 15.6% girls), could have a higher motivational effect, increasing daily PA and leading to a rise in the numbers of days when this recommendation is met.

Regardless of the specific daily step count recommendation, it is necessary to be aware of and respect geographic, socioeconomic, educational, national, and health specifics and sport preferences. The environment in Central Europe can be considered still acceptably walkable for adolescents, in spite of drawbacks related to transportation infrastructure and urbanism, such as heavy traffic, speeding, and lack of pedestrian-friendly crossings [58]. Traditionally high rates of adolescents involved in organised leisure-time PA in Poland [30] and the Czech Republic [22] as well as in Eastern Europe (e.g. Estonia, Lithuania) [21] are also a positive factor.

As there is a linear relationship between level of PA and adolescents’ health status [59], physically active adolescents suffer from fewer psychosocial symptoms [60], and as adolescents’ step counts decline as they grow older, less active adolescents are at high future risk of several diseases and medical conditions. At least it is positive, that the present study did not find a decrease in PA (expressed by steps/day) in group with the lowest PA level over the 8-year monitoring. School administration, in cooperation with parents and leisure-time institutions, including amateur sport clubs, should focus on less physically active adolescents in a similar manner to other socially marginalised and health-endangered populations, putting emphasis on the improvement of adolescents’ physical literacy [61, 62] and more efficient use of information technologies for positive changes in their lifestyle [44, 63]. In particular, prevention of cardiovascular diseases [9, 64] and mental health promotion [65] need to be prioritised. PA trends in representative samples of children and adolescents observed using simple indicators such as daily step counts are also essential in Central European countries. Awareness of these trends, as exists for example in Canada [66], is crucial for the development of efficient health, school, and educational policies.

### Strength and limitations

The eight-year monitoring period using the same methods under the consistent lead of the same research team is a strength of the present study. Achieving this will be more and more difficult in future studies, however, because of the rapid development and promotion of wearables and mobile technologies. The composition of weekly PA in adolescents with different level of PA, with special attention paid to adolescents at health risk, is much scarcer. The education effects of the research are inspiring for the adolescent physical literacy field because of the options for data analysis and feedback for participants provided directly by the Indares application. The main limitations of the present study include intentional quota sampling based only on type of school, where student teachers had their teaching practice, and size of the city. Due to the relatively small number of participants in each year of the research (182 to 253 per year), it was impossible to determine time trends in daily step counts. Moreover, we did not collect data on socioeconomic status or race/ethnicity. Even though Poland is a country with relatively small income inequalities (Gini coefficient = 0.28) [67] and mostly homogeneous population [68], lack of these nuisance variables could have affected our findings. Similarly, possession of more specific data on respondents PA (e.g. extracurricular sport or physical education lessons) could have better explained the weekly composition of adolescents’ PA.

It is likely that the findings on the weekly composition of adolescents’ PA are closely related to their education activities. Lower differences in step counts between groups with varying overall level of PA are perhaps related to increased education demands after the weekend. Adolescents’ higher PA on Fridays has been repeatedly observed throughout Central Europe (Czech Republic, Poland and Slovakia) [20], and is probably linked to the end of the school week education process and subsequent decrease in school-related stress and school commitments. Traditional family activities on Sundays and preparation for school might be a factor in why adolescents’ PA is low on Sundays. Therefore, future research should focus on associations between weekly PA composition in adolescents by their overall level of PA and demands of the education process at various types of schools.

## Conclusions

The present study confirms that differences in composition of weekly PA between adolescents with lower level of PA and those with higher level of PA are statistically non-significant. The adolescents with lower level of PA are most physically active on Fridays (boys 8409 and girls 8591 steps/day) and least physically active on Sundays (boys 5449 a girls 5694 steps/day), with less than 5% of them meeting the 11,000 steps/day recommendation. This is the same also in the group with higher overall level of PA. The largest difference between groups with lower and higher level PA was observed on Saturdays, while the smallest difference was found on Mondays. Thus, it is desirable to focus on promotion of PA in the less active portion of adolescents on Mondays. This could result in better compensation for lower PA at weekends and support ‘bridging the gap’ between those with lower and higher levels of PA in the following days. It might be promising that we did not observe a decrease of PA among the adolescents with the lowest level of PA over the 8-year study period.

## Data Availability

Written request could be sent to the corresponding author.
